# Supplementary material for: Structure–Property Relationships for Fluorinated and Fluorine-Free Superhydrophobic Crack-Free Coatings
Source: Polymers (Basel). 2024 Mar 24;16(7):885. doi: 10.3390/polym16070885 (PMC11013294; doi:10.3390/polym16070885)
Supplement: Supplementary file 1 [file polymers-16-00885-s001.zip › polymers-2884995-supplementary.pdf]

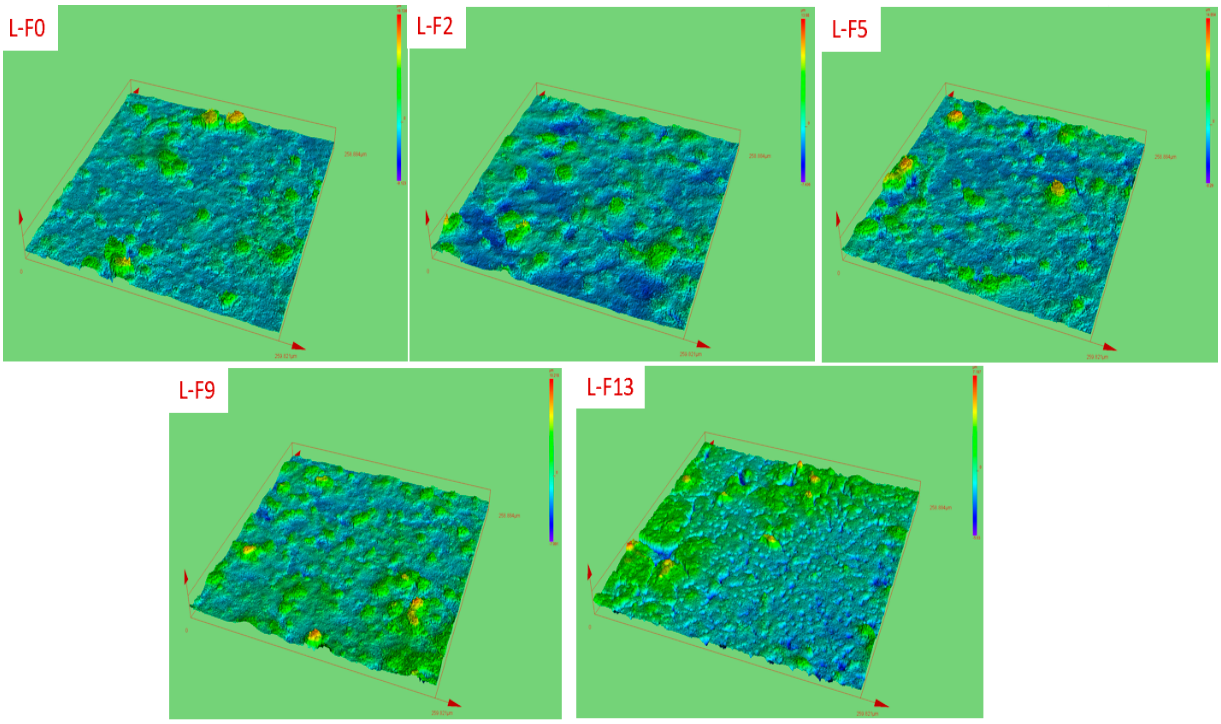

Figure S1. Confocal laser microscopy 3D images of L-F series coatings.

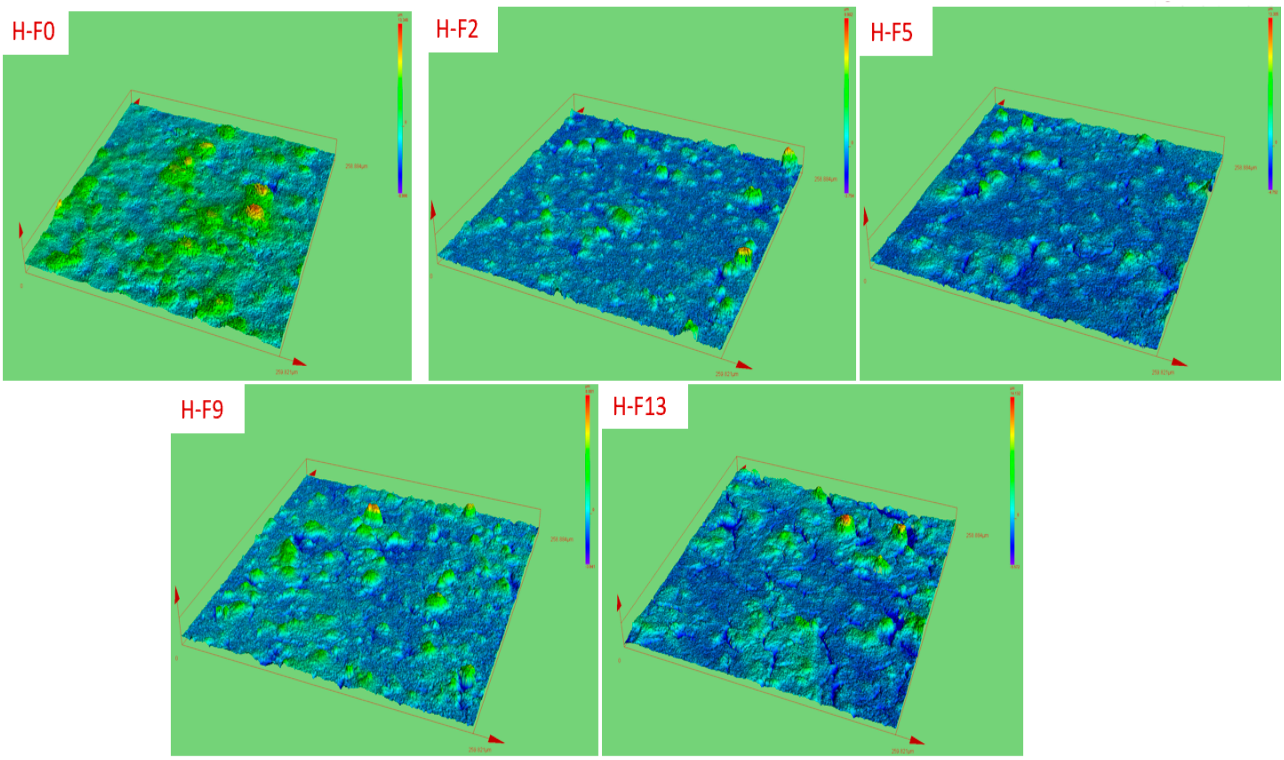

Figure S2. Confocal laser microscopy 3D images of H-F series coatings.

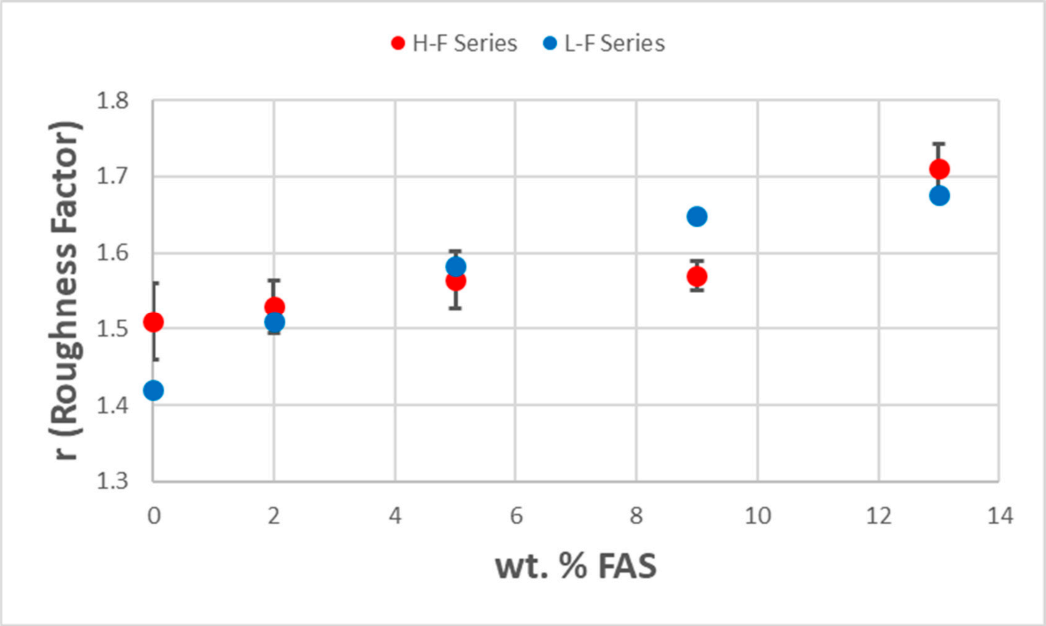

Figure S3. The Relationship of r with FAS content and particle loading

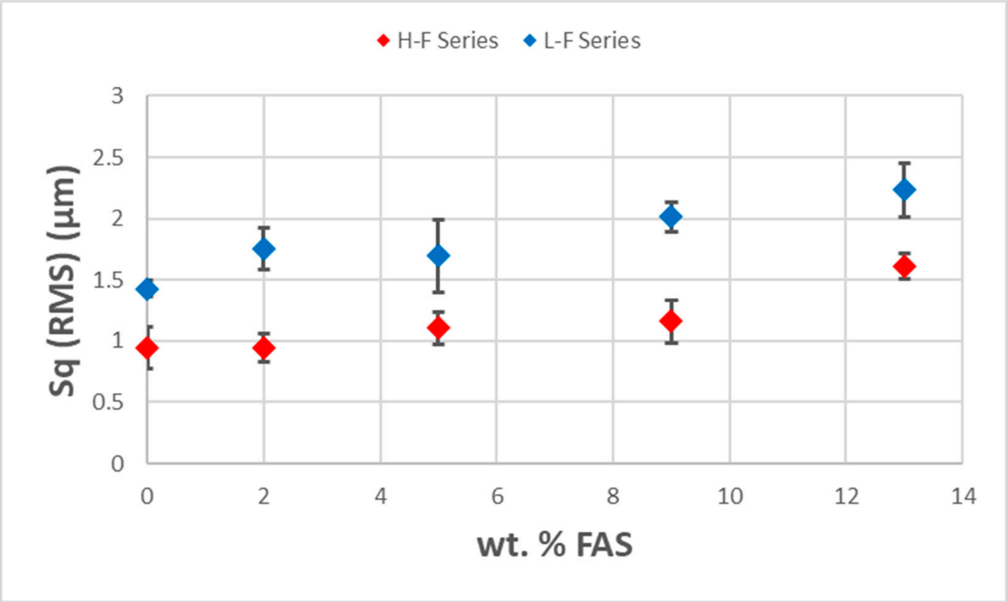

Figure S4. The Relationship of Sq with FAS content and particle loading
